# Treatment outcome of children with persistent Diarrhoea admitted to an Urban Hospital, Dhaka during 2012–2013

**DOI:** 10.1186/s12887-017-0896-7

**Published:** 2017-06-12

**Authors:** Mustafa Mahfuz, Mohammed Ashraful Alam, Shoeb Bin Islam, Nurun Nahar Naila, Mohammod Jobayer Chisti, Nur Haque Alam, Shafiqul Alam Sarker, Tahmeed Ahmed

**Affiliations:** Nutrition and Clinical Services Division (NCSD), International Centre for Diarrhoeal Disease Research, Bangladesh (icddr,b), 68, Shaheed Tajuddin Ahmed Sarani, Mohakhali, Dhaka, 1212 Bangladesh

**Keywords:** Persistent diarrhoea, Hospital acquired infection, Children, Icddrb, Dhaka

## Abstract

**Background:**

Despite availability of treatment guidelines, persistent diarrhoea (PD) has been a major contributor of diarrhoeal deaths in low and middle income countries. We evaluated the outcome of children under the age of 5 years who were treated for PD using management algorithm with locally available foods in a diarrhoeal disease hospital in Dhaka.

**Methods:**

We extracted retrospective data from electronic database for all the under-five children admitted for PD in the Longer Stay Ward and Intensive Care Unit of the Dhaka hospital at icddr,b between 2012 and 2013. Descriptive analysis was done to explore available baseline socio-demographic, nutritional, and co-morbid statuses, pathogens from stool isolates, duration of treatment, use of antibiotics, duration of hospital stay and treatment success rates. We sought to investigate above mentioned descriptive features in addition to associated factors with time to recover from PD using survival analysis with Cox proportional hazard model.

**Results:**

A total number of 426 children with a median age of 7.46 (inter-quartile range IQR; 5.39, 9.43) months were admitted for PD during the study period. Of these, 95% of children were recovered from PD and discharged from the hospital. The median duration of treatment response was 6 (IQR 4, 9) days. The case fatality rate was 1.17%. Multivariate analysis among the children of 6 months or less showed that the rate of recovery from PD was 57% lower in children with severe stunting compared to those without severe stunting (HR 0.43, 95% CI 0.22, 0.88, *p* < 0.05), 42% lower in children with severe wasting (HR 0.58, 95% CI 0.36, 0.95, *p* < 0.05), and 81% reduced in children who developed hospital acquired infection (HAI) compared to those without HAI (HR 0.19, 95% CI 0.06, 0.62, *p* < 0.05). Among the children who were more than 6 months old, age in months (HR 1.05, 95% CI 1.02, 1.09) and female gender (HR 1.41, 95% CI 1.09, 1.84) had better rates of recovery from PD (*p* < 0.05). Moreover, among children more than 6 months of age, HAI (HR 0.44, 95% CI 0.26, 0.75), and antibiotic use (HR 0.40, 95% CI 0.28, 0.56) were associated with impeded recovery rates from PD (*p* < 0.05).

**Conclusion:**

The treatment guideline for persistent diarrhoea patients followed at icddr,b Dhaka hospital was found to be successful and can be used in other treatment facilities of Bangladesh and other developing countries where any treatment algorithm for PD is unavailable. More emphasis is required to be given for the prevention of hospital acquired infection that may help to limit the use of antibiotic in order to enhance the recovery rate from PD.

**Electronic supplementary material:**

The online version of this article (doi:10.1186/s12887-017-0896-7) contains supplementary material, which is available to authorized users.

## Background

Diarrhoea remains one of the most common causes of child death [1]. Despite the improvement in the management of diarrhoea using oral rehydration solution, intravenous fluid and zinc, diarrhoea is still responsible for a death toll of around 522,000 deaths per year [2]. More than half (80%) of all the child deaths from diarrhoea occur in African and South-East Asian region including India, Nigeria, Congo, Afghanistan, Pakistan, Ethiopia and Bangladesh [3, 4]. Most of the diarrhoeal diseases are acute and last less than 7 days. Hence, when this acute phase extends to 14 days or more, it is termed as persistent diarrhoea [5]. A recent analysis concluded that persistent diarrhoea (PD) has been responsible for 32–62% diarrhoea associated deaths of young children in low- and middle-income countries [6]. Sixty percent of PD occurs before 6 months and 90% below 1 year of age [7]. Although in Bangladesh highest proportion of deaths occur due to acute diarrhoea, PD accounted for more than 25% of deaths among children aged 1–4 years and 40% of them were found malnourished [6]. Factors believed to be associated with PD are mucosal injury, delayed repair of mucosal damage, and host susceptibility; all of which are strongly influenced by malnutrition [8]. Moreover, several studies showed that malnutrition, younger age, lack of breastfeeding, infection, inappropriate use of antibiotics are factors associated with the development of PD [9, 10]. Due to multifaceted etiology, proper diagnosis and treatment is often warranted for quick recovery from such episodes. In addition, higher cost of treatment and high case fatality rates reiterate PD as an important Public health problem [11]. To explore PD management outcomes, we analyzed the hospital records (electronic data base) of all the children who were admitted to Dhaka Hospital of icddr,b with PD between 2012 and 2013.

## Methods

### Study design

This is a retrospective chart analysis where data were extracted from electronic database *(SHEBA*) of hospital records of Dhaka Hospital of icddr,b.

Study site: The Dhaka Hospital of icddr,b is located at Dhaka city, the capital of Bangladesh. This facility was established in 1962 to treat patients with diarrhoeal diseases. At present, more than 140,000 diarrhoeal patients receive treatment from this hospital in a year [9].

#### Study population

All the children admitted with PD to the Longer Stay Unit (LSU) and Intensive Care Unit (ICU) of Dhaka hospital diagnosed between January 2012 and December 2013 were eligible for this study. Available data include admission documents, treatment records, follow-up and discharge information. Patient information used in this analysis include duration of diarrhoea and vomiting, stool consistency, feeding history, breast feeding status, history of previous illness, dehydration status, treatment history, dietary treatment, antibiotic use, clinical outcomes, complications, available blood and stool microbiology test results and also socio-demographic and anthropometric data.

#### Patient management

**Fig. 1:**
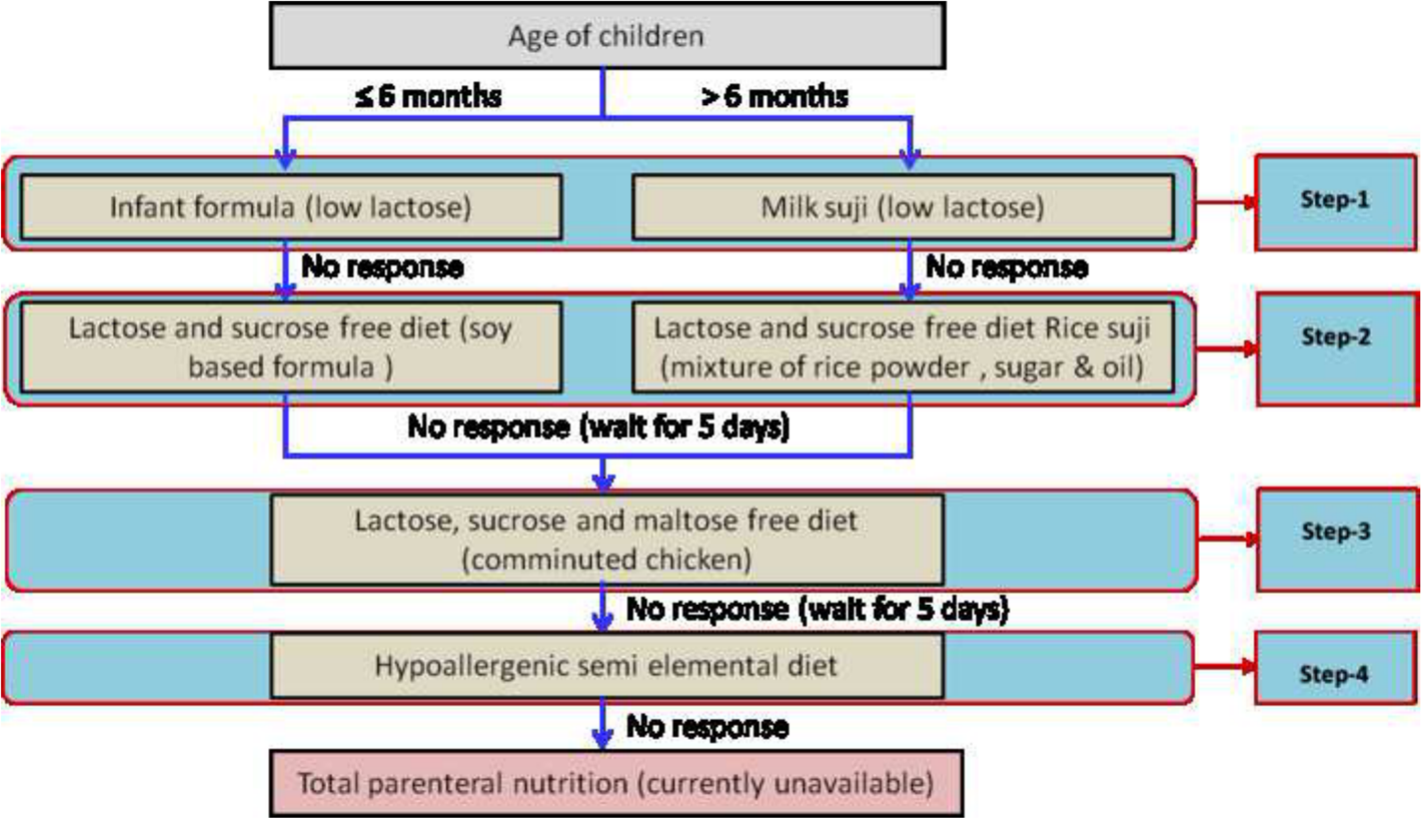
Treatment algorithm of International Centre for Diarrhoeal Research Centre, Bangladesh for children with persistent diarrhoea

At icddr,b persistent diarrhoea was managed according to the hospital’s PD management protocol that consists of dietary management algorithm with locally available foods. It includes correction of dehydration, infection control, dietary management, micronutrient supplementation and treatment of complications, if any. Rehydration was done using oral rehydration solution for children with ‘some’ dehydration and intravenous (IV) fluid for those with severe dehydration. IV fluid was also used in children with ‘some’ dehydration having persistent vomiting or high purging. The use of IV fluids in children with severe acute malnutrition (SAM) and severe dehydration was managed by icddr,b guidelines for the management of SAM [12]. Early diagnosis and treatment of infection including extra-intestinal infection was done through routine microscopy and culture of stool, and urine samples, if indicated. The dietary management was performed using the dietary protocol illustrated in this manuscript (Fig. 1).

#### Operational definitions

Diarrhoea was defined as three or more episodes of loose or watery stool in the last 24 h. Persistent diarrhoea was defined as any diarrhoea with or without blood, which began acutely and lasted for 14 days or more (i.e. without any diarrhoea free period of more than 48 h). Hospital acquired infection (HAI) was defined as any new infection that occurred in a patient during hospitalization, after 48 h of admission and was not present or incubating at the time of admission. Invasive diarrhoea was defined as presence of visible blood in stool or >20 pus cell/HPF with any number of RBC in routine microbiological examination of stool. A patient was considered as clinically cured if there was no diarrhoea or passage of soft or formed stool as documented by physician by direct visual inspection of stool passed in the bucket placed beneath the patient’s bed with a central hole (cholera cot) over the period of last 24 h. Recovery time was calculated as number of hours had passed after the start of dietary intervention until the passage of last liquid stool.

## Statistical analysis

All data were analyzed using STATA software (Version 13.1; StataCorp, College Station, Texas, USA). Data distribution was checked for normality by histogram and Q-Q plot. Considering difference in protocol all data were separately analyzed for children 6 months or less and 6–59 months of age. Percentage was calculated for categorical data. Medians and inter-quartile ranges, or mean and standard deviation were calculated for continuous data. Normality distributed data were compared by Student’s t-test and Mann-Whitney test was used for comparison of data that were not normally distributed. To explore the factors associated with time to recovery from PD, survival analysis with Cox proportional hazard model was carried out for both the treatment groups separately. Bivariate associations between each independent variable with persistent diarrhoea recovery rate were determined using unadjusted Cox proportional hazards models and variables associated with persistent diarrhoea recovery rate at the level of *p* < 0.2 were included in the multivariable model. Strength of association was determined by calculating hazard ratio (HR) and their 95% confidence intervals (CIs). A probability of less than 0.05 was considered statistically significant.

## Result

Demographic and clinical characteristics of all the study children are presented in Table 1. A total number of 426 children under the age of 5 years were admitted with Persistent Diarrhoea (PD) in a time period of 2012 to 2013. Median (inter-quartile range) age of the children was 7.46 (5.39, 9.43) months and 32% were 6 months or less during admission. Among the children 34.51% were female. Sixty nine percent children were breastfed, 82.21% used supply water for drinking and 88.60% children were immunized as per EPI schedule for Bangladesh. Among the children admitted with PD, 31.69% were 6 months or less and the remaining children were of more than 6 months (Table 1). The case fatality rate (CFR) was 1.17% for all the children admitted with PD during the years from 2012 to 2013. The CFR was 1.48% for the children of 6 months or less and 1.03% for older children.

**Table 1:** Demographic and laboratory characteristics of children admitted with persistent diarrhoea

	Age group		All children
	≤ 6 months (*n* = 135)	> 6 months (*n* = 291)	*N* = 426
Age in month, Mean ± SD	4.27 ± 1.22	9.75 ± 3.84	8.01 ± 4.13
Age in month, Median (IQR)	4.50 (3.48, 5.29)	8.84 (7.29, 36.93)	7.46 (5.39, 9.43)
Child sex, Female	40/135 (29.63%)	107/291 (36.77%)	147/426 (34.51%)
Drinking water, Supply water	71/86 (82.56%)	174/212 (82.08)	245/298 (82.21%)
Residence type, Slum	3/86 (3.49%)	7/215 (3.26%)	10/301 (3.32%)
Immunization			
As per EPI schedule	79/95 (83.16%)	193/212 (91.04%)	272/307(88.60%)
Incomplete	13/95 (13.68%)	17/212 (8.02%)	30/307 (9.77%)
Not done	3/95 (3.16%)	2/285 (0.94%)	5/307 (1.63%)
Weight in kg, Mean ± SD	5.34 ± 1.50	7.10 ± 1.39	6.55 ± 1.65
Height in cm, Mean ± SD	61.05 ± 4.93	69.32 ± 4.92	66.69 ± 6.25
Weight for age z score, Mean ± SD	−2.30 ± 1.67	−1.95 ± 1.47	−2.06 ± 1.54
Height for age z score, Mean ± SD	−1.26 ± 1.55	−1.13 ± 1.46	−1.17 ± 1.49
Weight for height z score, Mean ± SD	−1.85 ± 1.54	−1.76 ± 1.45	−1.79 ± 1.47
Still breastfeeding	77/109 (70.64%)	174/255 (68.24%)	251/364 (68.96%)
Severe underweight	44/133 (33.08%)	67/290 (23.10%)	111/423 (26.24%)
Severe stunting	18/134 (13.43%)	27/287 (9.41%)	45/421 (10.69%)
Severe wasting	29/131 (22.14%)	54/284 (19.01%)	83/415 (20.00%)
Associate clinical condition			
Abscess	0/129 (0%)	2/285 (0.70%)	2/414 (0.48%)
Amoebiasis	0/129 (0%)	1/285 (0.35%)	1/414 (0.24%)
Hospital acquired infection	6/129 (4.65%)	21/285 (7.37%)	27/414 (6.52%)
Pneumonia	10/129 (7.75%)	41/285 (14.39%)	51/414 (12.32%)
Septicaemia	3/129 (2.33%)	6/285 (2.11%)	9/414 (2.17%)
Tuberculosis	1/129 (0.78%)	0/285 (0%)	1/414 (0.24%)
Typhoid	1/129 (0.78%)	0/285 (0%)	1/414 (0.24%)
URTI	14/129 (10.85%)	17/285 (5.96%)	31/414 (7.49%)
UTI	9/129 (6.98%)	25/285 (8.77%)	34/414 (8.21%)
Invasive diarrhea	8/122 (6.56%)	19/263 (7.22%)	27/385 (7.01%)
Dehydration	63/135 (46.67%)	135/285 (46.39%)	198/426 (46.48%)
WBC status, Abnormal	23/130 (17.69%)	37/282 (13.12%)	60/412 (14.56%)
Neutrophil status^a^			
Normal (40–75)%	54/130 (41.54%)	134/282 (47.52%)	88/412 (45.63%)
Low <40%	75/130 (57.69%)	141/282 (50%)	216/412 (52.43%)
High >75%	1/130 (0.77%)	7/282 (2.48%)	8/412 (1.94%)
Lymphocyte status^a^			
Normal (20–45)%	36/130 (27.69%)	98/282 (34.75%)	134/412 (32.52%)
Low <20%	2/130 (1.54%)	11/282 (3.90%)	13/412 (3.16%)
High >45%	92/130 (70.77%)	173/282 (61.35%)	265/412 (64.32%)
Urine pus cells, Abnormal	7/79 (8.86%)	26/183 (14.21%)	33/262 (12.60%)
Urine protein, Abnormal	45/79 (56.96%)	90/183 (49.18%)	135/262 (51.53%)
Number of pathogen detected			
0	121/135 (89.63%)	259/291 (89%)	380/426 (89.20%)
1	14/135 (10.37%)	30/291 (10.31%)	44/426 (10.33%)
2	0/135 (0%)	2/291 (0.69%)	2/426 (0.48%)
Aeromonas detected	1/135 (0.74%)	1/291 (0.34%)	2/426 (0.47%)
Campylobacter detected	9/135 (6.67%)	12/291 (4.12%)	21/426 (4.93%)
Plesiomonas shigelloides detected	0/135 (0%)	1/291 (0.34%)	1/426 (0.23%)
Salmonella detected	2/135 (1.48%)	5/291 (1.72%)	7/426 (1.64%)
Shigella detected	1/135 (0.74%)	5/291 (1.72%)	6/426 (1.41%)
Vibrio cholerae detected	1/135 (0.74%)	10/291 (3.44%)	11/426 (2.58%)
Antibiotic used	99/135 (73.33%)	231/291 (79.38%)	330/426 (77.46%)

^a^Bain BJ. The peripheral blood smear. In: Goldman L, Schafer AI, eds. *Cecil Medicine*. 24th ed. Philadelphia, Pa: Saunders Elsevier; 2011:chap 160.

Severe malnutrition was common, as 26.24% of all the children were severely underweight (weight-for-age z-score < −3 SD of WHO growth standard), 10.69% were severely stunted (height/length-for-age z-score < −3 SD), and 20.00% of children were severely wasted (weight-for-height/length z-score < −3 SD). Nearly half of the children were dehydrated as recorded by clinical examination during hospital admission which was similar across both the age groups (46.67% for ≤6 months old and 46.39% for children of 6–59 months old). Major concurrent clinical conditions include severe acute malnutrition (SAM) (20.00%), pneumonia (12.32%), hospital acquired infection (HAI) (6.52%), invasive diarrhoea (7.01%), urinary tract infection (UTI) (8.21%), upper respiratory tract infection (URTI) (7.49%), and septicemia (2.17%). SAM was the most common associated clinical condition across both the age groups. After SAM, URTI, pneumonia, invasive diarrhoea and UTI were more common among the children of 6 months or less. Whereas, pneumonia, UTI and HAI were the second to fourth most common co-morbidities among children who were more than 6 months old (Table 1). Enteric pathogens were isolated from 10.80% of all stool samples. However, multiple pathogens were present only in 4.35% of all culture positive stool samples.

Using the hospital protocol that included dietary treatment algorithm, 92.59% children ≤6 months and, 96.22% of children aged >6 months were recovered from PD and discharged from the hospital. Median days for treatment response were 5 days for 6 months or less age group and 6 days for the age group of older than 6 months. The median hospital stay was 7 days for all the children (median, inter-quartile range: 7, 5–11 days) which was similar for both the age groups. Regarding treatment response to each dietary step, 54.73% children were recovered during step-1 of dietary treatment with low lactose formula. The proportion was 65.04% for the children of 6 months or less and 50.18% for the children aged more than 6 months. In the step-2, 38.06% of children (30.08% 6 months or less, 41.58% more than 6 months) were recovered with lactose and cow’s milk free diets (soy based formula or rice suji). The remaining children received diet mentioned in step-3 and proportion of children recovered from PD were 4.07% for the age group 6 months or less and 8.24% for the age group of more than 6 months. Only one child included in the age group of 6 months or less required hypo allergic diet (step-4). Antibiotic was prescribed for 77.46% of all children and requirement was more among the older children (6 months or less vs. more than 6 months, 73.33% vs. 79.38%) (Table 2).

**Table 2:** Persistent diarrhoea recovery, hospital stay and treatment response

	Age group		All children
	≤ 6 months (*n* = 135)	> 6 months (*n* = 291)	*N* = 426
PD recovered	125/135 (92.59%)	280/291 (96.22%)	405/426 (95.07%)
Treatment response days, Median (IQR)	5 (3, 8)	6 (4, 10)	6 (4, 9)
Hospital stay days, Median (IQR)	7 (4, 9)	8 (5, 12)	7 (5, 11)
Treatment response diet step			
Step-1	80/123 (65.04%)	140/279 (50.18%)	220/402 (54.73%)
Step-2	37/123 (30.08%)	116/279 (41.58%)	153/402 (38.06%)
Step-3	5/123 (4.07%)	23/279 (8.24%)	28/402 (6.97%)
Step-4	1/123 (0.81%)	0/279 (0%)	1/402 (0.25%)

**Fig. 2:**
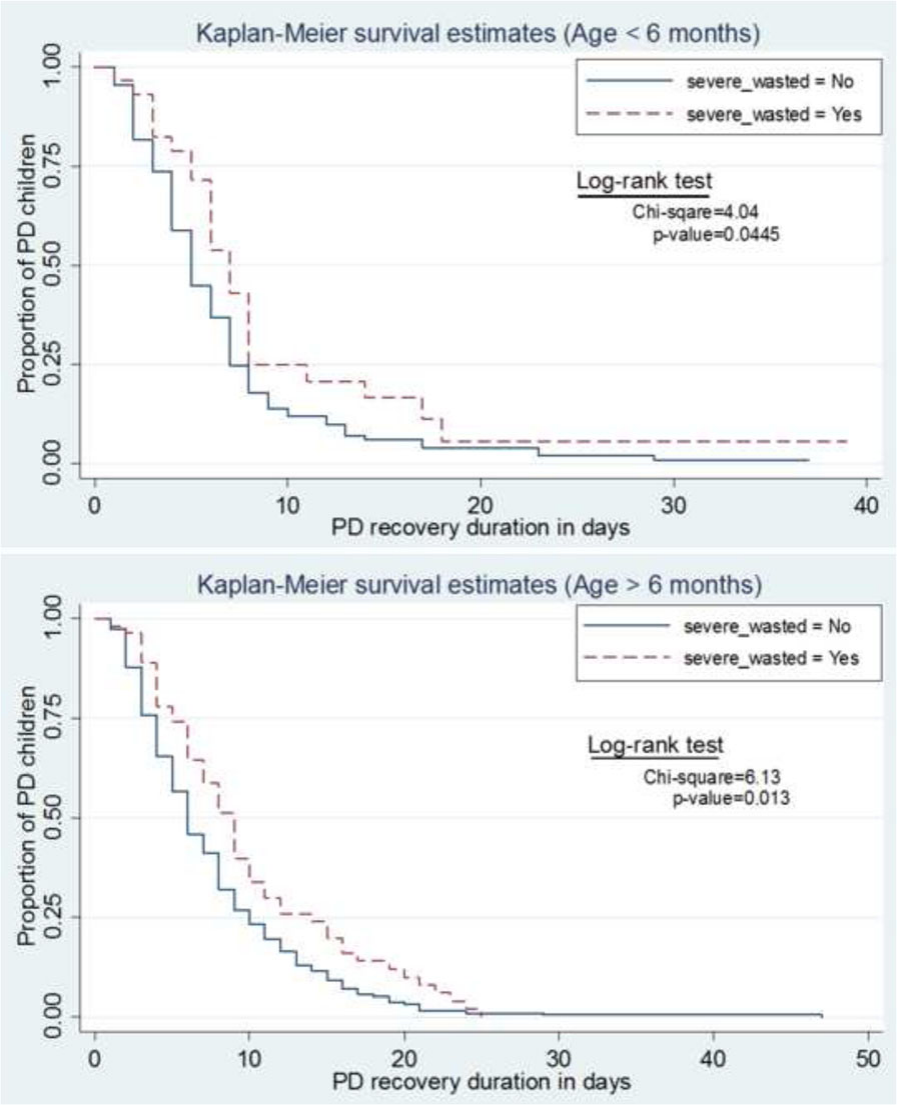
Kaplan-Meier survival estimates showing longer duration of recovery from PD in severely wasted children compared to non-severely wasted children

To explore the factors associated with the rate of recovery from PD, survival analysis with Cox proportional hazard model was carried out for both the age groups separately. In bivariate analysis, for the age group 6 months or less, the rate of recovery from PD was 54% less in severe stunted compared to non severe stunted children, and 84% less among infants who developed HAI, and 54% less in children with pneumonia compared to infants without any co-morbid condition. The rate of recovery from PD was 79% more among children with neutropenia than the children with normal neutrophil count (*p* < 0.05). For children aged >6 months, the rate of persistent diarrhoea recovery was 32% more in female compared to male and 48% more in neutropenia than the children with normal neutrophil counts (Table 3). On the other hand, the rate of recovery was 30% less in severe wasted children compared to non severe wasted children, 22% less in children with dehydration; 60% less in children who developed HAI and, 34% less in children who had pneumonia . Antibiotic use was associated with reduced rate of recovery from PD compared to children without antibiotic in more than 6 months age group (HR 0.39, 95% CI 0.29, 0.53) (Table 3). Kaplan-Meier survival estimates showed that PD recovery time were significantly higher in children with severe wasting in both the age groups (Fig. 2).

**Table 3:** Estimates of hazard ratios and 95% confidence intervals of persistent diarrhoea recovery

Characteristic	Children aged ≤6 month				Children aged >6 month			
	Unadjusted HR (95% CI)	*p*-value	Adjusted HR^a^ (95% CI)	*p*-value	Unadjusted HR (95% CI)	*p*-value	Adjusted HR^a^ (95% CI)	*p*-value
Age in month	0.96 (0.83, 1.11)	0.569			1.04 (1.01, 1.07)	0.004	1.05 (1.02, 1.09)	0.003
Child sex, Female	0.98 (0.67, 1.44)	0.911			1.32 (1.03, 1.68)	0.028	1.41 (1.09, 1.84)	0.009
Still breastfeeding	0.86 (0.58, 1.25)	0.425			0.97 (0.76, 1.25)	0.829		
Drinking water, Supply water	0.91 (0.58, 1.42)	0.680			1.67 (0.84, 1.61)	0.347		
Residence type, Slum	0.83 (0.30, 2.27)	0.718			0.92 (0.54, 1.58)	0.774		
Severe underweight	0.80 (0.55, 1.17)	0.245			0.86 (0.65, 1.14)	0.302		
Severe stunting	0.46 (0.27, 0.80)	0.006	0.43 (0.22, 0.83)	0.011	1.02 (0.68, 1.52)	0.926		
Severe wasting	0.67 (0.43, 1.03)	0.070	0.58 (0.36, 0.95)	0.030	0.70 (0.52, 0.95)	0.022	0.76 (0.54, 1.06)	0.107
Dehydration	1.07 (0.75, 1.53)	0.696			0.78 (0.62, 0.99)	0.043	0.95 (0.73, 1.23)	0.684
Co-morbidity condition								
None	Ref				Ref		Ref	
Hospital acquired infection	0.16 (0.05, 0.48)	0.001	0.20 (0.07, 0.58)	0.003	0.40 (0.25, 0.66)	<0.001	0.43 (0.27, 0.67)	0.001
Pneumonia	0.46 (0.23, 0.94)	0.034	0.63 (0.28, 1.40)	0.256	0.66 (0.45, 0.96)	0.032	0.73 (0.49, 1.08)	0.115
URTI	1.44 (0.78, 2.67)	0.250	2.30 (1.17, 4.53)	0.016	1.25 (0.74, 2.11)	0.396	1.64 (0.95, 2.84)	0.077
UTI	0.69 (0.34, 1.43)	0.324	1.15 (0.54, 2.49)	0.714	0.95 (0.61, 1.48)	0.818	1.14 (0.70, 1.87)	0.597
Others^b^	0.90 (0.32, 2.52)	0.842	1.91 (0.62, 5.90)	0.262	0.73 (0.36, 1.51)	0.402	0.80 (0.38, 1.70)	0.563
WBC status, Abnormal	1.26 (0.79, 2.00)	0.335			1.08 (0.76, 1.53)	0.668		
Neutrophil status^c^								
Low (<40%)	1.79 (1.22, 2.61)	0.003	1.56 (0.88, 2.75)	0.125	1.48 (1.16, 1.89)	0.001	1.30 (0.88, 1.93)	0.191
High (>70%)	2.28 (0.31, 16.74)	0.417	6.20 (0.32, 120.21)	0.228	0.69 (0.32, 1.49)	0.349	1.58 (0.42, 5.95)	0.496
Lymphocyte status^c^								
Low (<20%)	1.52 (0.36, 6.40)	0.568	0.46 (0.06, 3.63)	0.458	0.65 (0.35, 1.22)	0.180	0.57 (0.19, 1.67)	0.302
High (>45%)	1.45 (0.96, 2.19)	0.077	1.00 (0.54, 1.87)	0.993	1.35 (1.04, 1.74)	0.022	1.19 (0.79, 1.78)	0.411
Invasive diarrhoea	1.07 (0.52, 2.21)	0.853			1.11 (0.69, 1.80)	0.659		
Urine pus cells, Abnormal	0.81 (0.46, 1.41)	0.450			0.81 (0.58, 1.14)	0.222		
Urine protein, Abnormal	0.79 (0.55, 1.12)	0.187	0.89 (0.60, 1.31)	0.560	0.79 (0.63, 1.00)	0.054	1.18 (0.90, 1.53)	0.228
Any pathogen detected	0.96 (0.53, 1.74)	0.883			0.75 (0.51, 1.09)	0.134		
Antibiotic used	0.81 (0.53, 1.25)	0.345			0.39 (0.29, 0.53)	<0.001	0.40 (0.28, 0.56)	<0.001

^a^Only variables associated with persistent diarrhoea recovery rate at the level of *p* < 0.2 in bivariate analyses were included in adjusted model

^b^Others means Abscess/ Amoebiasis/ Septicaemia/ Tuberculosis/ Typhoid

^c^Bain BJ. The peripheral blood smear. In: Goldman L, Schafer AI, eds. *Cecil Medicine*. 24th ed. Philadelphia, Pa: Saunders Elsevier; 2011:chap 160.

In children of 6 months or less, multivariate analysis showed that severe stunting was associated with 57% reduced rate of recovery from PD than those who did not have severe stunting (HR 0.43, 95% CI 0.22, 0.83) and, children with severe wasting had 42% reduced rate of recovery from PD (HR 0.58, 95% CI 0.36, 0.95). HAI was independently associated with 81% reduced rate of recovery from PD than those who did not have any co-morbid condition (HR 0.19, 95% CI 0.06, 0.62). For the children aged more than 6 months, development of HAI (HR 0.44, 95% CI 0.26, 0.75) and, antibiotic use (HR 0.40, 95% CI 0.28, 0.56) were associated with reduced rate of recovery from PD. Additionally, age (HR 1.05, 95% CI 1.02, 1.09) and female gender (HR 1.41, 95% CI 1.09, 1.84) were associated with increased rates of recovery from PD (*p* < 0.05) (Table 3). For better understanding, an additional table describing the average recovery rates according to the different variables assessed that underlies the hazard ratios is included [see Additional file 1: Average recovery rates of different variables assessed underlying the hazard ratios].

## Discussion

Malnutrition is considered as one of the most crucial factors for development of PD and moreover, recurring episodes of diarrhoea in PD usually lead to malabsorption and subsequent malnutrition [13]. Nutritional (macro- and micronutrient) management is therefore very important for the management of PD [14, 15]. Published reports indicate that dietary treatment consists of locally available inexpensive foods and micronutrient supplementation had tremendous success rate in the management of PD among children [16, 17]. However, the continuation of breastfeeding is considered to be one of the major components in the case management of PD for young children. Those children who are not breast fed for any reason or weaned to complementary food, low lactose or lactose free diet are provided [18]. Lactose-free liquid feedings reduce the duration and risk of treatment failure compared to lactose-containing liquid feedings in diarrhoea [17–21]. An international working group had developed and tested an algorithm through a multi country cohort for the treatment of PD nearly 20 years ago. This treatment algorithm was based on the use of locally available and affordable foods, vitamin and mineral supplementation, and the appropriate use of antibiotics for treating the associated infections [16]. A total of 460 children were treated with two different dietary regimes. The overall success rate of recovery from PD using this algorithm was 80% [16]. This study provided evidence about the role of simple clinical guidelines and use of locally available food for the successful short term treatment of PD. World Health Organization had been recommending this algorithm for the management of PD. But the protocol was implemented only in few places and hence, a policy is required to promote the use of this protocol in treatment of PD [6].

The Dhaka Hospital of International Centre for Diarrhoeal Disease Research, Bangladesh (icddr,b) is one of the largest diarrhoeal disease hospital in the world. Icddr,b has been following a treatment protocol for PD for more than two decades which is similar to WHO recommendation (Fig. 1). At icddr,b, two varieties of diets based on low lactose milk are provided to the children under the age of 5 years as dietary management of persistent diarrhoea. Infant formula (low lactose milk, sugar and oil, 68, kcal/100 ml) is offered to the children 6 months or less and children aged more than 6 months are provided with ‘*Milk suji’* (low lactose milk, rice powder, sugar and vegetable oil, 70 Kcal/100 g) (step-1). Lactose and cow’s milk protein free diet is given to those children who do not show any improvement with initial diet. At this stage, infants ≤6 months receive soy based diet (commercially available in different brands) and older children receive ‘*rice suji’*, a mixture of rice powder, egg white, sugar and oil (70 Kcal/100 g) (step-2). The children only receive the subsequent steps of diet only if they failed to improve after taking the diet of step 1. When the children do not respond to soy based diet or *rice-suji*, chicken based diet (minced chicken, glucose and oil, 60 kcal/100 g) is given to them (step-3). Moreover, the children who do not show any improvement with the above mentioned diet are prescribed with hypoallergic hydrolyzed formula (commercially available formula, Pregestimil, Mead Jonson) (step-4). Clinical judgment is the decisive factor to determine the duration of dietary treatment with any of the given dietary regime. However, a child is switched to the next step of dietary regime if no improvement is seen after 5 to 7 days of treatment with existing dietary regime.

Our study suggested that the current treatment protocol used by Dhaka Hospital of icddr,b has been successfully treating the children with PD at a recovery rate of 95%. The stepwise dietary modification using the locally available foods was the mainstay of the treatment. Treatment with milk based low lactose products (formula or milk suji) and lactose and cow’s milk free diets (soy-based formula or rice suji) resulted in more than 90% of recovery. Chicken based diet was required only for few children and hypoallergic semi elemental diet was required for only one child for the complete recovery from PD.

The median treatment response period of 6 days was also considered to be modest. However, the unavailability of similar data from other health facilities impeded the relative comparison of treatment efficiency. Multivariate analysis by Cox proportional hazard model showed that both the severe stunting and severe wasting were significantly associated with the reduction of treatment response rate from PD for the children of 6 months or less age groups. It is already known that children with PD had a greater chance of becoming stunted compared to those without PD [22]. Moreover, acute malnutrition is one of the most important predictor of prolongation of PD [9]. Neutropenia is a significant factor found to be associated with faster recovery from PD in bivariate analysis. We do not know the actual reason for the positive role of neutropenia in quick recovery from PD. Concurrent infection was very common among the children who were admitted with PD. Therefore, the overwhelming infections that used up neutrophils faster than their production may be responsible for this relative abundance of neutropenia [23]. Similar feature was also observed in other countries [24]. Moreover, previous studies from the same setting had shown that children with acute diarrhoea with altered immune response and elevated plasma cytokines were associated with subsequent development of PD [25, 26]. However, there is no explanation why neutropenia as a result of concurrent infection can improve PD outcome. Neutropenia is one of the important visible markers of severe infection and probably for that the children having neutropenia were treated aggressively. As a result, they responded well. As recovery from PD is proportionately related to the recovery from concomitant infection, this might be one of the probable explanations for this observation. A large number of associated clinical conditions including SAM, pneumonia, URTI and, HAI were recorded in this study. Therefore, this analysis reiterates the fact that infection is common among the children with PD.

Enteropathogens attributable to PD have been changed over the last 2 decades with a decline in *Shigella* spp. and *Vibrio cholera* and increased isolation of rotavirus from the stool samples [9]. Shigellosis is well recognized for the pathogenesis of PD and its reduction has been considered as a decisive factor for low prevalence of PD in Dhaka hospital [27, 28]. In 2010, no *Shigella* was isolated from the children with PD at icdd,b hospital [9], which is however, observed in current analysis among the children of PD. Previous studies conducted at icddr,b argued that enteroaggregative *Escherichia coli* has been a major contributor for PD and other common pathogens for PD were *Aeromonas*, *Klebsiella* [29], and *Cryptosporidium* [30]. Rotavirus and *E coli* isolation is not routinely done in Dhaka hospital; therefore, we could not show any information regarding this. Another study conducted in Dhaka hospital reported that *Campylobacter*, rotavirus, *V.cholera* and *Salmonella* are the common pathogens responsible for hospital acquired infection [31]. Hospital acquired infection was found to be an important variable that has been consistently associated with reduced rate of recovery from PD in all multivariate analyses. The presence of *Campylobacter* spp., *Vibrio cholera* and, *Salmonella* in the stool culture of current analysis might be due to high level of HAI among the admitted children; an issue needs to be addressed. Although isolation of relatively high *Campylobacter* spp. in PD can be explained by the recent evidences acquired from the MAL-ED cohort, where, *Campylobacter* exhibited one of the highest burdens of community diarrhoea among the children less than 2 years [32].

Antibiotic use was high among these children and this was also revealed in previous studies [30]. As icddr,b is a diarrhoeal disease hospital and children with PD admitted in this hospital with concurrent morbidities of infectious origin, antibiotics were prescribed as per different treatment guidelines. Our analyses showed that use of antibiotic had significantly impeded the rate of recovery from PD in older children. Use of antibiotics prior to hospital admission and irrational use of antibiotics are known risk factors of persistent diarrhoea [9]. Considering this current observation that antibiotics were hindering the recovery rates, this is now imperative to have an evaluation of antibiotic use in PD [9]. Moreover, prevention of HAI may also reduce the need for long term use of antibiotics.

There had been a number of limitations in the current analysis. The data were extracted from the hospital records. The information was limited and some of the important variables including stool volume, stool colour, history of vomiting, name of antibiotic etc. required for this type of analysis were absent. Current analysis used the data of all the children who were presented with PD during admission. We did not consider children who developed PD after hospitalization. Lack of long term follow-up is another limitation of this analysis. Objective diagnosis of complete recovery from PD was a drawback. Since most of the children admitted to this hospital with acute diarrhoea were treated with antibiotics and antimicrobial has a proven role in duration of PD, this had been a major shortfall. Lack of adequate data on antibiotic use, particularly limited information about the type and combination of antibiotics had also prevented us from defining the attribution of individual antibiotic or combinations on recovery from PD. Moreover, hospital records showed that five children who had been admitted to this hospital with PD were died during 2012 to2013. The case fatality rate was similar to the current trend of PD as observed by Das et al. [9].

## Conclusion

Despite availability of well validated treatment algorithm, a substantial number of children are still dying from PD. Recently WHO reported on the poor improvement in the coverage of proper treatment of diarrhoea since 2000. The report showed that more than 60% of children with diarrhoea in developing countries did not receive the recommended treatment. This finding highlights on the significance of a more comprehensive input towards persistent diarrhoea [33]. Nonetheless, this analysis from hospital records of 2 year (2012–2014) provided the information on treatment outcome of all the children below 5 years old who were admitted with PD in one of the largest diarrhoeal disease hospitals in the world. With proper management of hospital acquired infections and judicious use of antibiotics, this simple dietary management algorithm with locally available food ingredients along with the simple guidelines followed at icddr,b Dhaka hospital can be used for better treatment outcomes in children with PD in the other treatment facilities where any treatment algorithm for PD is still unavailable.

## Additional file

Additional file 1: Average recovery rates of different variables assessed underlying the hazard ratios. (PDF 67 kb)

## Abbreviations

ALRI: Acute lower respiratory tract infection
CI: Confidence interval
HAI: Hospital acquired infection
HR: Hazard ratio
icddr,b: International Centre for Diarrhoeal Disease Research, Bangladesh
ID: Invasive diarrhoea
PD: Persistent diarrhoea
SAM: Severe acute malnutrition
UTI: Urinary tract infection
WHO: World Health Organization
