# Supplementary material for: Treatment outcome of children with persistent Diarrhoea admitted to an Urban Hospital, Dhaka during 2012–2013
Source: BMC Pediatr. 2017 Jun 12;17:142. doi: 10.1186/s12887-017-0896-7 (PMC5469056; doi:10.1186/s12887-017-0896-7)
Supplement: Additional file 1: — Average recovery rates of different variables assessed underlying the hazard ratios. (PDF 67 kb) [file 12887_2017_896_MOESM1_ESM.pdf]

**Additional file 1**

| Characteristic | Children age ≤6 months | | | | Children age >6 months | | | |
|---|---|---|---|---|---|---|---|---|
| | Number of children | Days of follow-up | Number of recovered children | Recovery rate per child-year | Number of children | Days of follow-up | Number of recovered children | Recovery rate per child-year |
| Child sex | | | | | | | | |
| Male | 95 | 641 | 88 | 50.11 | 184 | 1565 | 179 | 41.75 |
| Female | 40 | 260 | 37 | 51.94 | 107 | 715 | 101 | 51.56 |
| Still breastfeeding | | | | | | | | |
| No | 42 | 242 | 39 | 58.82 | 93 | 725 | 91 | 45.81 |
| Yes | 93 | 659 | 86 | 47.63 | 198 | 1555 | 189 | 44.36 |
| Severe underweight | | | | | | | | |
| No | 90 | 567 | 85 | 54.72 | 223 | 1711 | 217 | 46.29 |
| Yes | 45 | 334 | 40 | 43.71 | 68 | 569 | 63 | 40.41 |
| Severe stunting | | | | | | | | |
| No | 116 | 687 | 109 | 57.91 | 262 | 2059 | 253 | 44.85 |
| Yes | 19 | 214 | 16 | 27.29 | 29 | 221 | 27 | 44.59 |
| Severe wasting | | | | | | | | |
| No | 106 | 661 | 100 | 55.22 | 237 | 1758 | 228 | 47.34 |
| Yes | 29 | 240 | 25 | 38.02 | 54 | 522 | 52 | 36.36 |
| Dehydration | | | | | | | | |
| No | 72 | 496 | 65 | 47.83 | 156 | 1112 | 150 | 49.24 |
| Yes | 63 | 405 | 60 | 54.07 | 135 | 1168 | 130 | 40.63 |
| Drinking water | | | | | | | | |
| Tube Well | 25 | 161 | 24 | 54.41 | 49 | 397 | 44 | 40.45 |
| Supply water | 110 | 740 | 101 | 49.82 | 242 | 1883 | 236 | 45.75 |
| Residence type | | | | | | | | |
| None slum | 131 | 865 | 121 | 51.06 | 277 | 2160 | 266 | 44.95 |
| Slum | 4 | 36 | 4 | 40.56 | 14 | 120 | 14 | 42.58 |

| Characteristic | Children age ≤6 months | | | | Children age >6 months | | | |
|---|---|---|---|---|---|---|---|---|
| | **Number of children** | **Days of follow-up** | **Number of recovered children** | **Recovery rate per child-year** | **Number of children** | **Days of follow-up** | **Number of recovered children** | **Recovery rate per child-year** |
| Co-morbidity condition | | | | | | | | |
| None | 43 | 232 | 42 | 66.08 | 91 | 641 | 90 | 51.25 |
| Hospital acquired infection | 6 | 120 | 5 | 15.21 | 21 | 298 | 20 | 24.50 |
| Pneumonia | 10 | 98 | 10 | 37.24 | 41 | 410 | 40 | 35.61 |
| URTI | 14 | 64 | 14 | 79.84 | 17 | 105 | 17 | 59.10 |
| UTI | 9 | 67 | 9 | 49.03 | 25 | 185 | 25 | 49.32 |
| Others** | 5 | 27 | 4 | 54.07 | 9 | 74 | 8 | 39.46 |
| WBC status | | | | | | | | |
| Normal | 111 | 772 | 103 | 48.70 | 253 | 2001 | 244 | 44.51 |
| Abnormal | 24 | 129 | 22 | 62.25 | 38 | 279 | 36 | 47.10 |
| Neutrophil status | | | | | | | | |
| Normal | 54 | 472 | 47 | 36.35 | 134 | 1218 | 130 | 38.96 |
| Low | 75 | 412 | 75 | 66.44 | 141 | 937 | 137 | 53.37 |
| High | 1 | 5 | 1 | 73.00 | 7 | 84 | 7 | 30.42 |
| Lymphocyte status | | | | | | | | |
| Normal | 36 | 297 | 31 | 38.10 | 98 | 877 | 95 | 39.54 |
| Low | 2 | 12 | 2 | 60.83 | 11 | 139 | 11 | 28.88 |
| High | 92 | 580 | 90 | 56.64 | 173 | 1223 | 168 | 50.14 |
| Monocyte status | | | | | | | | |
| Normal | 96 | 690 | 90 | 47.61 | 222 | 1727 | 214 | 45.23 |
| Low | 2 | 19 | 2 | 38.42 | 3 | 26 | 3 | 42.12 |
| High | 32 | 180 | 31 | 62.86 | 57 | 486 | 57 | 42.81 |
| Invasive diarrhea | | | | | | | | |
| No | 126 | 830 | 117 | 51.45 | 271 | 2143 | 262 | 44.62 |
| Yes | 9 | 71 | 8 | 41.13 | 20 | 137 | 18 | 47.96 |
| Urine pus cells | | | | | | | | |

| Characteristic | Children age ≤6 months | | | | Children age >6 months | | | |
|---|---|---|---|---|---|---|---|---|
| | Number of children | Days of follow-up | Number of recovered children | Recovery rate per child-year | Number of children | Days of follow-up | Number of recovered children | Recovery rate per child-year |
| Normal | 121 | 784 | 111 | 51.68 | 248 | 1889 | 239 | 46.18 |
| Abnormal | 14 | 117 | 14 | 43.68 | 43 | 391 | 41 | 38.27 |
| Urine protein | | | | | | | | |
| Normal | 59 | 372 | 57 | 55.93 | 141 | 1011 | 138 | 49.82 |
| Abnormal | 76 | 529 | 68 | 46.92 | 150 | 1269 | 142 | 40.84 |
| Any pathogen detected | | | | | | | | |
| No | 121 | 800 | 113 | 51.56 | 259 | 1978 | 249 | 45.95 |
| Yes | 14 | 101 | 12 | 43.37 | 32 | 302 | 31 | 37.47 |
| Antibiotic used | | | | | | | | |
| No | 36 | 205 | 28 | 49.85 | 60 | 259 | 56 | 78.92 |
| Yes | 99 | 696 | 97 | 50.87 | 231 | 2021 | 224 | 40.46 |

**Others means Abscess/ Amoebiasis/ Septicaemia/ Tuberculosis/ Typhoid
